# Adrenomedullin promotes angiogenesis in epithelial ovarian cancer through upregulating hypoxia-inducible factor-1α and vascular endothelial growth factor

**DOI:** 10.1038/srep40524

**Published:** 2017-01-16

**Authors:** Yi Zhang, Yang Xu, Jian Ma, Xiaoyan Pang, Mei Dong

**Affiliations:** 1 Department of Gynecology, First Affiliated Hospital of China Medical University, Shenyang 110001, Liaoning, China; 2 Department of Gynecology, Shenyang Forth People’s Hospital, Shenyang 110001, Liaoning, China; 3 Department of Geriatrics, No. 401 Hospital of PLA, Qingdao 266071, China

## Abstract

Adrenomedullin (ADM) is a multi-functional peptide related to many kinds of tumors. This study was aimed to investigate the role of ADM on angiogenesis in epithelial ovarian cancer (EOC) and its possible mechanism. The expressions of ADM, vascular endothelial growth factor (VEGF), hypoxia-inducible factor-1α (HIF-1α) and CD34 were examined by immunohistochemistry staining. The relationship among ADM, HIF-1α, VEGF and micro-vessel density (MVD) was assessed in 56 EOC tissues. CAOV3 cells were stably transfected with pcDNA-ADM (plasmid overexpressing ADM gene) or pRNA-shADM (small interfering RNA for ADM gene). Real-time PCR and western blot analysis were performed to detect the expressions of HIF-1α and VEGF. The MTT, transwell migration assay and *in vitro* tube formation analysis were used to evaluate the proliferation, migration, and tube formation ability of human umbilical vein endothelial cells (HUVECs) which were pretreated with ADM or ADM receptor antagonist ADM22-52. Our findings showed that ADM expression was positively correlated with the expressions of HIF-1α, VEGF or MVD in EOC. ADM upregulated expression of HIF-1α and VEGF in CAOV3 cells. ADM promoted HUVECs proliferation, migration and tube formation. In conclusion, ADM was an upstream molecule of HIF-1α/VEGF and it promoted angiogenesis through upregulating HIF-1α/VEGF in EOC.

Epithelial ovarian cancer (EOC) is one of the most common causes of death from all cancers among women and the leading cause of death from gynecological malignancies^1^. Due to occult onset and easily metastasis, nearly 60–70% of EOC patients are diagnosed at an advanced stage^2^. Strong evidences suggest that tumor angiogenesis has vital prognostic significance in advanced ovarian cancer^3^, but the specific mechanism is still largely unknown.

Adrenomedullin (ADM), a multi-functional peptide, initially identified in human pheochromocytoma^4^. ADM is widely expressed in many kinds of tumors, such as breast, colon, thyroid, prostate, lung and ovarian neoplasms^5^, and involves in tumor angiogenesis, inhibiting apoptosis, immune escape and other processes which seem to have an important role in tumor biological behaviors^6^.

Angiogenesis is a process by which new microvessels sprout from existing vessels. A great number of proangiogenic and antiangiogenic regulators have been identified^7^ and the disruption of the balance eventually leads to angiogenesis. Vascular endothelial growth factor (VEGF) is the most pivotal proangiogenic regulator of angiogenesis^8^. Hypoxia-inducible factor-1 (HIF-1) is a transcription factor and HIF-1α is the oxygen-regulated subunit that determines HIF-1 activity. Currently, HIF-1 is proved to be associated with the transcriptions of many genes, including ADM and VEGF^9^. In the regulatory parts of the ADM gene, there are at least 20 putative binding sites for the transcription factor complex HIF-1^10^, and HIF-1 is the key regulator of hypoxia-inducible genes, such as VEGF^11^. ADM, VEGF, and HIF-1 are all associated with tumor angiogenesis, but whether ADM modulates angiogenesis and interacts with VEGF and HIF-1 in EOC remains unknown.

In the present study, we analyzed the role of ADM in angiogenesis *in vitro*, and investigated the relationship among ADM, VEGF and HIF-1α in EOC. We aimed to probe the possible mechanism of ADM on angiogenesis in EOC.

## Results

### Positive Correlation among the Expressions of ADM, HIF-1α, VEGF and CD34 in EOC

**Figure 1:**
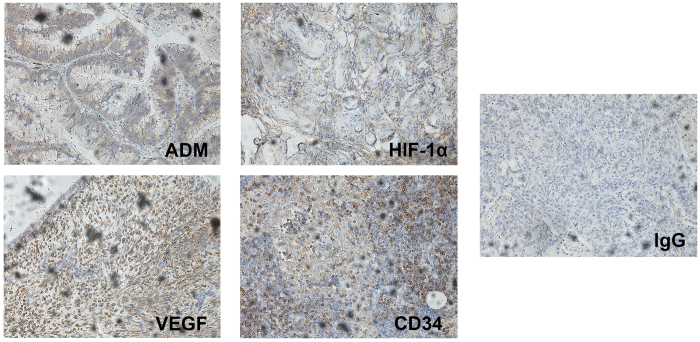
Immunohistochemical staining for ADM, HIF-1α, VEGF and CD34 showing positive in EOC tissue (×200).

ADM, HIF-1α, VEGF, and CD34 were mainly expressed in the cytoplasm and membranes of EOC cells, seldom in nuclei (Fig. 1). Among 56 EOC tissues, integrated optical density (IOD) was used to quantitatively analyze the immunostaining intensity. A positive correlation was found among ADM, HIF-1α, VEGF, and CD34 (Table 1).

### Positive Correlation between the Expressions of ADM and MVD in EOC

To detect the correlation between ADM expression and tumor angiogenesis, we assessed the micro-vessel density (MVD), the marker of angiogenesis, in the 56 EOC tissues. MVD was defined as the mean number of CD34^+^ vessels per section. A significantly higher MVD in EOC was found compared to normal ovarian tissues (*p* = 0.02). MVD in EOC was associated with degree of differentiation (*p* = 0.014), but not with age and Federation International of Gynecology and Obstetrics (FIGO) stage (Table 2). EOC tissues with higher integrated optical density (IOD) of ADM expression had a significantly higher MVD value. Likewise, EOC tissues with higher IOD of VEGF or HIF-1α expression showed similar phenomena. The correlations of ADM, HIF-1α or VEGF expression with MVD were positive (Table 3).

### Expressions of HIF-1a and VEGF was regulated by ADM in CAOV3 cells

**Figure 2:**
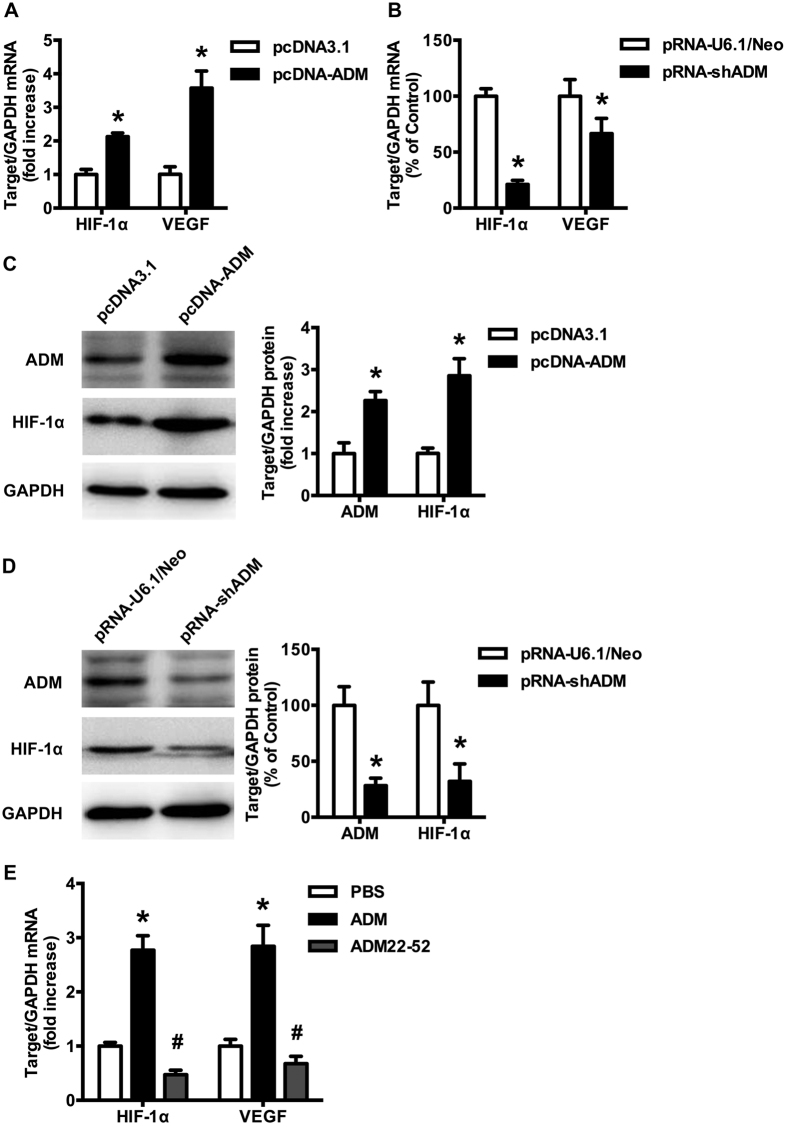
Effect of ADM on HIF-1α and VEGF expressions. HIF-1α and VEGF mRNA expressions were examined by real-time PCR. The relative expression levels of target genes were normalized to GAPDH. The expression level of HIF-1α was examined by western blot. Empty vectors were used as control. **(A)** ADM overexpression in CAOV3 cells upregulated the mRNA expressions of HIF-1α and VEGF. Data are presented as the means ± SEM of triplicate experiments. *p < 0.05 versus control. **(B)** ADMsiRNA in CAOV3 cells downregulated the mRNA expressions of HIF-1α and VEGF. Data are presented as the means ± SEM of triplicate experiments. *p < 0.05 versus control. **(C)** ADM overexpression upregulated the protein expressions of HIF-1α in CAOV3 cells. **(D)** ADMsiRNA downregulated the protein expressions of HIF-1α in CAOV3 cells. Right panel: quantification of protein levels. **(E)** ADM upregulated expressions of HIF-1α and VEGF and ADM22-52 downregulated expressions of VEGF and HIF-1α in CAOV3 cells. CAOV3 cells were treated with ADM (10 nM) or ADM22-52 (1 nM) for 24 h. Data are presented as the means ± SEM of triplicate experiments. *p < 0.05 versus control, ^#^*p* < 0.05 versus ADM treatment.

To determine whether ADM regulates HIF-1α and VEGF transcriptional activation in EOC, CAOV3 cells were transfected with pcDNA-ADM (plasmid overexpressing ADM) or pRNA-shADM (small interfering RNA for ADM) to upregulate or knockdown the ADM gene, then the expression levels of HIF-1α and VEGF in CAOV3 cells were examined using real-time PCR and western blot. As shown in Fig. 2A, when ADM gene was upregulated in CAOV3 cells with plasmid pcDNA-ADM, HIF-1α and VEGF mRNA expressions were enhanced greatly (*p* = 0.001 and 0.006, respectively), especially VEGF more than 3 times higher compared with control group. On the contrary, when ADM gene was silenced with shRNA (Fig. 2B), VEGF and HIF-1α mRNA expressions were inhibited significantly (*p* = 0.000 and 0.046, respectively). The protein expression of HIF-1α showed the similar change (Fig. 2C,D). To further test this effect of ADM, CAOV3 cells were treated with ADM (10 nM) or ADM22-52, the antagonist of ADM, (1 nM) for 24 h, and the mRNA expression levels of HIF-1α and VEGF were examined using real-time PCR. The result showed that ADM increased VEGF and HIF-1α mRNA expressions in CAOV3 cells (*p* = 0.000 and 0.009, respectively), and ADM22-52 decreased the expressions of HIF-1α and VEGF (*p* = 0.002 and 0.038, respectively, Fig. 2E). These data showed that either exogenous or endogenous ADM could upregulate the gene expressions of VEGF and HIF-1α in CAOV3 cells, ADM22-52 or knockdown ADM gene, showed the contrary effect, indicating that ADM was an upstream regulator of HIF-1α and VEGF.

### ADM Promotes HUVECs Proliferation, Migration and Tube Formation *in Vitro*

**Figure 3:**
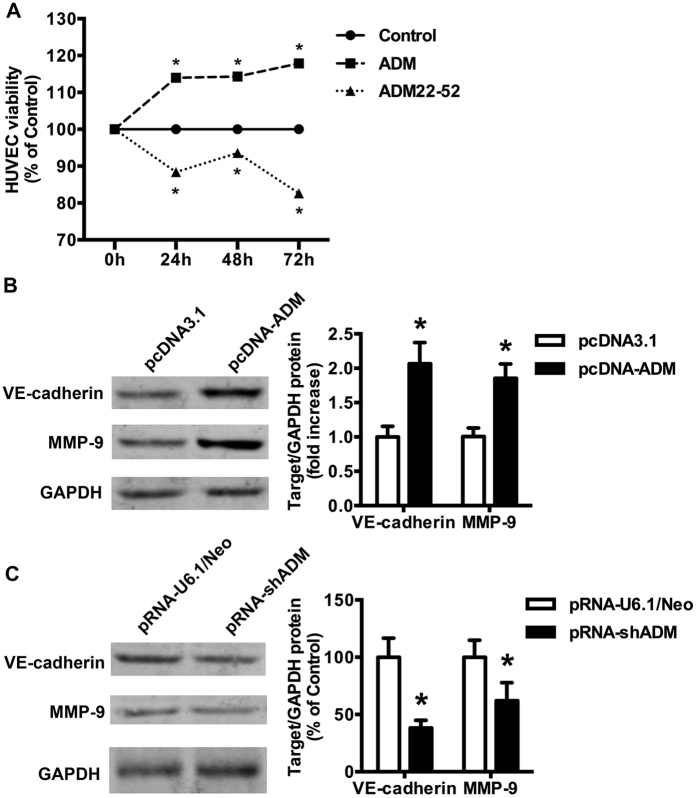
Effects of ADM and ADM22-52 on angiogenesis. **(A)** Effects of ADM and ADM22-52 on HUVECs proliferation. HUVECs were treated with ADM (100 nM) or ADM22-52 (1 nM) for 24 h, 48 h and 72 h, respectively. Cells were counted by MTT. Data are means ± SEM of triplicate experiments. *p < 0.05 versus control. **(B)** ADM overexpression upregulated the protein expressions of VE-adherin and MMP-9 in CAOV3 cells. **(C)** ADMsiRNA downregulated the protein expressions of VE-adherin and MMP-9 in CAOV3 cells. Right panel: quantification of protein levels.

**Figure 4:**
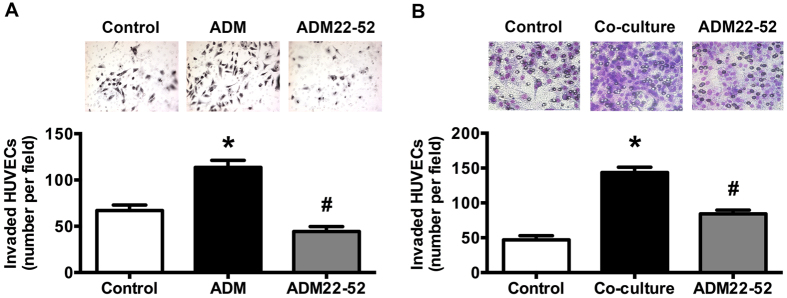
ADM and ADM22-52 influenced HUVECs migration *in vitro*. **(A)** HUVECs were seeded on transwell with ADM (100 nM) or ADM22-52 (1 nM) for 24 h. PBS was used as control. **(B)** HUVEC were seeded on upper chamber of transwell, co-cultured with CAOV3 cells in lower chamber, and treated with or without ADM22-52 (1 nM) for 24 h. Upper panel: the migration ability of HUVECs was assessed by Transwell migration assay (×400). Lower panel: cells counted in five microscopic fields (means ± SEM). *p < 0.05 versus control, ^#^*p* < 0.05 versus ADM treatment or co-culture treatment.

**Figure 5:**
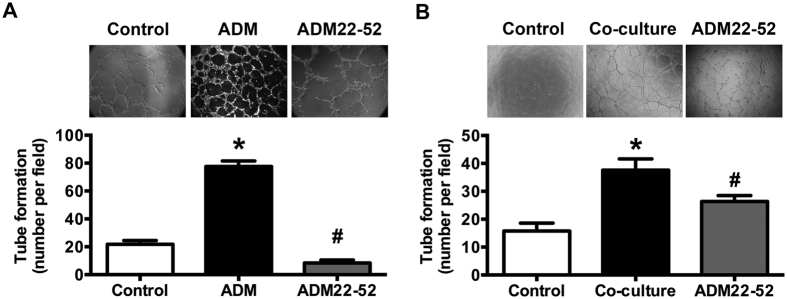
ADM and ADM22-52 influenced HUVECs tube formation *in vitro*. **(A)** HUVEC were seeded on Matrigel with ADM (100 nM) or ADM22-52 (1 nM) for 24 h. **(B)** HUVEC were seeded on Matrigel co-cultured with CAOV3 cells, and treated with or without ADM22-52 (1 nM) for 24 h. Upper panel: tube-like structures photographs. Lower panel: tube-like structures counted in five microscopic fields (means ± SEM). *p < 0.05 versus control, ^#^*p* < 0.05 versus ADM treatment or co-culture treatment.

To determine the direct angiogenic effect of ADM, various angiogenic properties were studied using human umbilical vein endothelial cells (HUVECs). MTT assay was performed to evaluate the ADM effect on HUVECs proliferation. Figure 3A indicated that ADM promoted HUVECs proliferation directly in a time-dependent manner (24 h, 48 h, 72 h, *p = *0.000, 0.049, and 0.025, respectively) and ADM22-52 inhibited HUVECs proliferation also in a time-dependent manner (24 h, 48 h, 72 h, *p = *0.047, 0.002, and 0.021, respectively). ADM resulted in a promotion of 133.26% in 72 h treatment. Early at the 24 h treatment, the inhibition of ADM22-52 could be observed. So, in the following studies we chose 24 h as a representative time point of ADM22-52. Interference of ADM expression also regulated the protein levels of VE-cadherin and MMP-9, which plays the important role in tumor angiogenesis (Fig. 3B,C). Therefore, a transwell chamber system was employed to determine the effects of ADM on the migration of HUVECs. As shown in Fig. 4A, ADM (100 nM for 24 h) increased HUVECs migration across the transwell membrane (*p* = 0.000). Contrarily, ADM22-52 (1 nM for 24 h) decreased the invasive potential of HUVECs (*p* = 0.000) co-culture with CAOV3 cells, indicating ADM secreted from CAOV3 cells is crucial for HUVECs migration (Fig. 4B). We also evaluated the effect of ADM on the formation of capillary-like tube structures by plating HUVECs on Matrigel. The result showed that ADM promoted tube like structure formation of cultured HUVECs, and ADM22-52 inhibited this effect (*p* = 0.000, Fig. 5A,B).

## Discussion

The present study demonstrated that ADM promoted tumor angiogenesis through upregulating HIF-1α/VEGF in EOC *in vitro*, ADM/HIF-1α/VEGF might be a new signaling pathway playing a role in this process.

Strong evidence suggested that ADM involved in physiological and pathological angiogenesis in some tissues and cell lines. ADM induces the growth of human endometrial microvascular endothelial cells and regulates the angiogenesis in female reproductive tract^12^. In uterine leiomyomas and renal tumors, the expression of ADM mRNA is correlated with vascular density^13^,^14^. In xenografted tumor models utilizing human endometrial, breast, pancreatic tumor cell lines or human glioblastoma cells, ADM overexpressing transfectants increase the growth of blood vessels or vascular density^15^,^16^,^17^,^18^. VEGF and HIF-1α are potent inducer of angiogenesis and tumor growth^19^,^20^. Preclinical studies suggest that VEGF-mediated angiogenesis is important in initiating and mediating the growth of ovarian cancers^21^. HIF-1α is often upregulated in human cancers to regulate VEGF expression by binding to the hypoxia responsive element of VEGF promoter^22^.

In the present study, we investigated the relationship between ADM expression and MVD of 56 EOC tissues with immunohistochemical analysis, and also the relationship among ADM, VEGF and HIF-1α in EOC for the first time. We found the direct clues that ADM, VEGF and HIF-1α expressions were all positively related to MVD in EOC *in vivo*, which agreed with the results shown in renal tumors^14^ and uterine leiomyomas^13^. We also observed a positive correlation among ADM, VEGF and HIF-1α expressions in EOC *in vitro*, which is consistent with our previous report^23^. Higher level of ADM was closely correlated with expressions of HIF-1α and VEGF. These data indicated that ADM might induce HIF-1α/VEGF expression and contribute to angiogenesis in clinical EOC. The effect of ADM on angiogenesis may be bound up with VEGF and HIF-1α. Besides, we found that MVD in EOC was only associated with degree of differentiation, which was similar to ADM expression in EOC in our previous study^24^. We therefore considered that MVD could reflect the biological aggressiveness in EOC as ADM.

In order to further investigate the role of ADM/HIF-1α/VEGF on angiogenesis in EOC, we constructed plasmid pcDNA-ADM and pRNA-shADM to endogenously increase or decrease the ADM gene expression in EOC cell line CAOV3. Then we provided exogenous ADM and ADM22-52, the ADM antagonist, to CAOV3 cells. HIF-1α/VEGF expressions were examined by real time PCR and western blot. We found that ADM upregulated HIF-1α and VEGF expressions, and ADM22-52 downregulated HIF-1α and VEGF expressions in CAOV3 cells. These results suggested that ADM was an upstream molecule of HIF-1α/VEGF. HIF-1α and VEGF were important regulators in tumor angiogenesis, which were required for tumorigenesis and tumor development^25^,^26^. ADM may promote angiogenesis via upregulating HIF-1α/VEGF.

In addition, we treated HUVECs with ADM and ADM22-52 to determine the direct effect of ADM on angiogenesis. We observed that ADM promoted HUVECs proliferation, migration and tube formation *in vitro* while ADM22-52 inhibited these effects. These observations were consistent with the previous results^27^.

The angiogenic process is regulated by several “classic” factors, such as VEGF, HIF-1α and fibroblast growth factor-2 (FGF-2). These factors together with their receptors are currently the main targets against angiogenesis. Our results confirmed that ADM is an upstream molecule of HIF-1α/VEGF, so we suppose ADM may be involved in the regulation of other angiogenic “classic” factors to promote tumor angiogenesis. It was reported that HIF-1α induced the expression of ADM mRNA under normoxic and hypoxia conditions in the human ovarian carcinoma cell line OVCAR-3^10^, which was opposite to what we observed. Furthermore, recent observation^24^,^28^ proved that focal adhesion kinase (FAK) and ADM may play a cooperative role in EOC. Additional studies are needed to further demonstrate the role of ADM on EOC angiogenesis and the relationship between ADM and HIF-1α.

In summary, our present study showed that ADM was an upstream molecule of HIF-1α/VEGF, and promoted angiogenesis through upregulating HIF-1α/VEGF in EOC. Thus, ADM/HIF-1α/VEGF signaling pathway may be a possible therapeutic target in EOC in the future.

## Materials and Methods

### Cell Culture

Human EOC cell line-CAOV3 cells were cultured in RPMI1640 containing 10% fetal bovine serum (Thermo Scientific Hyclone, USA) and penicillin/streptomycin (100 U/ml). Cells were maintained at 37 °C in a humidified atmosphere of 5% CO_2_. When 80% confluent, cells were treated with ADM (100 nM, Phoenix Pharmaceuticals, USA) or ADM22-52 (1 nM, Phoenix Pharmaceuticals, USA) for 24 h.

Human umbilical vein endothelial cells (HUVECs) were freshly isolated as described previously^29^, from human umbilical veins of newborn obtained from a parturient at Shenyang 242 Hospital who gave written informed consent. HUVECs were routinely grown in DMEM (Gibco, USA) supplemented with 10% fetal bovine serum (Gibco, USA) and endothelial cell growth supplement (BD Biosciences, USA) at 37 °C and 5% CO_2_. In our experiments, only the first three passages of each HUVECs primary cultured were used.

### Tissue Samples

The studies have been approved by the ethical committee of China Medical Univarsity and have been performed in accordance with the ethical standards as laid down in the 1964 Declaration of Helsinki and its later amendments or comparable ethical standards. nformed consent was obtained from all subjects. For immunohistochemical analysis, EOCs (n = 56) were collected from surgical specimens originating from the First Affiliated Hospital of China Medical University between 2000 and 2008. The normal ovarian tissues were collected from 10 perimenopausal hysteromyoma patients who underwent hysterectomy and prophylactic adnexectomy. Clinical data were obtained from medical records. The tumors were staged according to FIGO guidelines and were single type of primary ovarian cancer without preoperative chemotherapy. The age of patients ranged from 44 to 66 years (median = 53.07). Of all the cases, 18 were FIGO I-II stage and 38 were FIGO III-IV stage.

### Immunohistochemical Staining

Unstained 4 μm paraffin sections from tissue sample were deparaffinized and rehydrated. The sections had been hematoxylin-and-eosin (HE) stained to confirm histological diagnosis by two pathologists according to the World Health Organization (WHO) classifications. All the sections were subjected to antigen retrieval by heating in Tris-EDTA buffer at pH 8.0 in an autoclave sterilizer for 2 min. Endogenous peroxidase activity was blocked with 3% hydrogen peroxide (H_2_O_2_) in methanol and non-specific binding sites were blocked with protein blocking solution (5% normal horse and 1% normal goat serum). Primary antibodies against ADM (1: 100 dilution, R&D, USA)/VEGF (1:200 dilution, Santa Cruz, CA)/HIF-1α (1:50 dilution, Santa Cruz, CA)/CD34 (1:100 dilution, DAKO Cytomation, Glostrup, Denmark) were added and sections were incubated over night at 4 °C. Then the sections were treated with secondary antibody and incubated with streptavidin-peroxidase (SP) complex (Maixin Biotechnology, Fujian, China) for 40 min at room temperature. Binding sites were visualized with 3, 3-diainobenzidine (DAB) (Maixin Biotechnology, Fujian, China) after 1 min incubation. Finally, sections were counterstained with hematoxylin, dehydrated with ethanol, fixed with xylene and mounted. Phosphate-buffered saline (PBS) was used instead of the primary antibodies for the negative control. For quantitative analysis of immunostaining intensity, integrated optical density (IOD) was employed to compute the relative value of each section. Digitally fixed images were analyzed at x200 magnification using an AxioImager A1 (Zeiss, Germany) light microscope equipped with an image analyzer (Image Pro Plus, Italy). IOD was calculated for arbitrary areas (20 arbitrary areas/samples, 1000 μm × 1500 μm) and each section analyzed with the same size.

Microvessel density (MVD) per section was measured using immunostaining with a CD34-monoclonal antibody. MVD was assessed according to the international consensus^30^. The entire section was scanned systematically at low magnification (×100) in order to identify the most intense areas of neovascularization (hotspots) within the tumor. After five hotspots areas with the highest number of capillaries and small venules were identified, microvessels were counted at high power magnification (×400), and the average in five fields was calculated.

### Plasmid Construction

ADM cDNA (aa154-711) was amplified by RT-PCR from the total RNA isolated from the CAOV3 cells. The two primers were: forward 5′-GGA TCC ATG AAG CTG GTT TCC ATC GC-3′, reverse 5′-GAA TTC CTA TAA CCT AGA GAC TCT GG-3′. The PCR products (576 bp) were inserted into the pcDNA3.1 (GeneScript, Piscataway, NJ, USA) vectors to form new vectors named pcDNA-ADM. The insert sequences were confirmed by DNA sequencing.

In our previous study, we had constructed short hairpin RNA (shRNA) targeting ADM gene *in vitro*^31^. The oligonucleotide shRNA based on the small interfering (siRNA) sequences were cloned into pRNA-U6.1/Neo (GeneScript, Piscataway, NJ, USA), named pRNA-shADM.

### Stably Transfected CAOV3 Cells

CAOV3 cells (5 × 10^4^) were seeded in 6-well tissue culture plates (BD Falcon, USA) and allowed to attach overnight to achieve 70% confluence at the time of transfection. 2 μg of purified plasmid DNA, encoding either vector alone, pcDNA-ADM or pRNA-shADM was transfected into cells using Lipofectamine TM 2000 (Carlsbad, CA) per manufacturer’s protocol. The cells were incubated for 24 h, and then 1 ml medium containing 20% serum was added to each well. The cells were cultured and screened in medium containing 10% serum and 400 μg/ml G418 for at least 3 weeks. Then, stable transfectants were formed. pcDNA3.1 or pRNAU6.1/neo vector was used as a control. The targeting sequences were validated by real-time PCR and western blot analysis.

### Real-time PCR

Total RNA was isolated from treated confluent CAOV3 cells using Trizol reagent according to the manufacturer’s instructions, resuspended in RNase-free water and stored at −80 °C. RNA concentration was measured by absorbance reading at 260 nm. Total RNA (2 μg) was reverse transcribed into cDNA using reverse transcription system (Promega, USA). The final cDNA product was stored at −20 °C. Real time PCR was carried out using SYBR Premix Ex Taq Kit (Takara, Tokyo, Japan) on Applied Biosystems 7500 (Foster City, CA, USA). The PCR reaction mixture of 20 μl contained 10 μl SYBR Premix Ex Taq (2 ×), 0.7 μl forword primer, 0.7 μl reverse primer and 2.0 μl cDNA. The forward and reverse PCR primers were summarized below (Supplementary Table 1). The reaction conditions were 50 °C for 2 min, 95 °C for 10 min, 95 °C for 30 sec and 60 °C for 30 sec for 50 cycles. The mRNA levels were normalized with respect to the levels of GAPDH in each sample.

### Western Blot

Total protein lysates were isolated from treated confluent CAOV3 cells using lysis buffer (0.01 mmol/l Tris-HCl, pH 7.6, 0.1 mmol/l NaCl, 0.001 mol/l EDTA, pH 8.0, 1 μg/ml Aprotinin, 100 μg/ml PMSF). Protein concentrations were determined by BCA Protein Assay Kit (PIERCE, Rockford, IL). Proteins were separated by 10% SDS-PAGE and then transferred to polyvinylidene fluoride membranes, which were then blocked for 2 h in 5% defatted milk in Tris-buffered saline containing Tween-20 (TBST, 10 mM Tris-HCl, 150 mM NaCl, 0.1% Tween-20). Membranes were then incubated for 2 h at room temperature with the following primary antibodies: ADM (1:1000, Phoenix, USA), HIF-1α (1:200, Transduction Laboratories, Heidelberg, Germany), VEGF (1:200, Phoenix, USA) and GAPDH (1:1000 Keygen Biotech, China). Primary antibodies were recognized by appropriate HRP-conjugated secondary antibodies (GE Healthcare Life Sciences, Piscataway, NJ) and visualized using the enhanced chemiluminescence detection system (ECL, Beyotime Institute of Biotechnology, China). The MF-ChemiBIS 3.2 Imaging System (DNR Bio-Imaging Systems, Israel) was used for image capture.

### MTT Assay

MTT assay was performed to evaluate the effect of ADM on HUVECs proliferation. HUVECs were seeded into 96-well plate at a density of 3 × 10^4^ cells/well. After incubating with FBS-free medium for 24 h, HUVECs were co-incubated with ADM (Phoenix Pharmaceuticals, USA) (100 nM) or ADM22-52 (Phoenix Pharmaceuticals, USA) (1 nM) or PBS (equal volume as control) for 24 h, 48 h and 72 h. Then, 20 μl of a sterile, filtered 3-(4, 5-dimethylthiazol-2-yl)-2, 5-diphenyltetrazolium bromide (MTT) solution (5 mg/ml) in PBS (pH 7.4) was added to each well and incubated for 4 h at 37 °C, which was followed by adding 150 μl dimethyl sulfoxide (DMSO) and incubating at 37 °C for an additional 10 min. Absorbance was read at 560 nm on a microplate reader.

### Migration Assay

For HUVECs migration assay, Transwell inserts (8 μm pore, Corning Costar Corp, Cambridge, MA, USA) were used as described^32^. At the top chambers, HUVECs were counted and resuspended in serum-free DMEM containing 0.1% BSA at a final concentration of 5 × 10^4^ cells/100 μl/well. The bottom chambers (600 μl) were filled with DMEM with 10% FBS. The HUVECs were co-incubated with PBS (as control), ADM (100 nM) or ADM 22-52 (1 nM) and allowed to invade for 24 h at 37 °C with 5% CO_2_. After incubation, the noninvasive cells that remained on the upper surface of the filter were removed by a cotton swab. The invaded cells were fixed and stained with 0.1% crystal violet, and then counted using a light microscope in 5 random fields per well.

### *In Vitro* Tube Formation Assays

The effect of ADM on angiogenesis *in vitro* was examined by tube formation assay. The wells of a 96-well plate were coated with 50 μl of ice-cold Growth Factor Reduced Matrigel (BD Bioscience, San Jose, CA) at 37 °C for 1 h. HUVECs were seeded at a density of 5 × 10^4^ cells per well in 200 μl complete culture medium containing ADM (100 nM) or ADM 22-52 (1 nM) or PBS (equal volume as control). After incubation for 24 h at 37 °C with 5% CO_2_, the cultures were photographed and the tube-like structures were evaluated.

### Statistical Analysis

Data were presented as the mean ± standard deviation (SD), and analyzed with one-way ANOVA or Student’s *t* test where applicable, by using SPSS (version 16.0, Chicago, IL, USA). Spearman rank correlation analysis was performed to examine the correlations among different variables. All the experiments were repeated at least three times. A *p* < 0.05 was defined as statistically significant.

## Additional Information

**How to cite this article**: Zhang, Y. *et al*. Adrenomedullin promotes angiogenesis in epithelial ovarian cancer through upregulating hypoxia-inducible factor-1α and vascular endothelial growth factor. *Sci. Rep.* **7**, 40524; doi: 10.1038/srep40524 (2017).

**Publisher's note:** Springer Nature remains neutral with regard to jurisdictional claims in published maps and institutional affiliations.

## Supplementary Material

Supplementary Table 1

## Figures and Tables

**Table 1:** Relationship of ADM, HIF-1α, VEGF and CD34 expression in EOC tissues.

	HIF-1α		VEGF		CD34	
	*r*	*p*	*r*	*p*	*r*	*p*
ADM	0.648	0.012	0.679	0.008	0.771	0.001
HIF-1α			0.578	0.03	0.718	0.004
VEGF	0.578	0.03			0.569	0.034
CD34	0.718	0.004	0.569	0.034		

**Table 2:** Relationship between MVD and clinicopathological features in EOC tissues.

		MVD		
Features		n	means ± SD	*p*
Age (years)	<50	18	40.17 ± 17.30	0.097
	≥50	38	39 ± 19.04	
FIGO stage	I–II	18	49 ± 17.88	0.094
	III–IV	38	32.38 ± 14.61	
Differentiation	High/Middle	24	27 ± 14.83	0.014
	Low	32	48.88 ± 13.62	

**Table 3:** Relationship of ADM, HIF-1α, VEGF and MVD in EOC tissues.

		ADM	HIF-1α	VEGF
MVD	*r*	0.711	0.568	0.537
	*p*	0.004	0.034	0.048
