# Supplementary material for: Adrenomedullin promotes angiogenesis in epithelial ovarian cancer through upregulating hypoxia-inducible factor-1α and vascular endothelial growth factor
Source: Sci Rep. 2017 Jan 16;7:40524. doi: 10.1038/srep40524 (PMC5238385; doi:10.1038/srep40524)
Supplement: Supplementary Table 1 [file srep40524-s1.pdf]

Adrenomedullin promotes angiogenesis in epithelial ovarian cancer through upregulating hypoxia-inducible factor-1α and vascular endothelial growth factor

Yi Zhang[1]*[&], Yang Xu[2]*, Jian Ma[3], Xiaoyan Pang[1], Mei Dong[2]

[1] Department of Gynecology, First Affiliated Hospital of China Medical University, Shenyang 110001, Liaoning, China;

[2] Department of Gynecology, Shenyang Forth People's Hospital, Shenyang 110001, Liaoning, China;

[3] Department of Geriatrics, No. 401 Hospital of PLA, Qingdao 266071, China

*These authors contributed equally to this work

[&]To whom correspondence should be addressed: Department of Gynecology, The First Affiliated Hospital of China Medical University, Shenyang 110001, Liaoning, China; E-Mail: syzi@163.com (Y.Z.); Tel./Fax: +86-24-8328-3510.

Suppl Table 1. The sequences of primers for real-time PCR

Gene name	Sequence of primers (5’-3’)
ADM	F：TCCCCCTATTTTAAGACGTGAATG<br>R：CATGCACACAAACACACTCACAT
HIF-1α	F：GGACAAGTCACCACAGGA<br>R：GGAGAAAATCAAGTCGTG
VEGF	F：GAAGGAGGAGGGCAGAATCATCAC<br>R：CACAGGATGGCTTGAAGATGTACTC
GAPDH	F：TGCATCCTGCACCACCAACTGCTTAG<br>R：TTCACCACCATGGAGAAGGC
